# Quantitative Assessment of the Association between rs2046210 at 6q25.1 and Breast Cancer Risk

**DOI:** 10.1371/journal.pone.0065206

**Published:** 2013-06-13

**Authors:** Xi Wu, Qing-Qing Xu, Liang Guo, Chuan-Ting Yu, Yu-Yu Xiong, Zhi-Yun Wei, Ran Huo, Sheng-Tian Li, Lu Shen, Jia-Min Niu, Lu Liu, Yi Lin, Lin He, Sheng-Ying Qin

**Affiliations:** 1 Bio-X Institutes, Key Laboratory for the Genetics of Developmental and Neuropsychiatric Disorders (Ministry of Education), Shanghai Jiao Tong University, Shanghai, P.R. China; 2 Shanghai Institutes of Genome Pilot and Human Health, Shanghai, China; 3 The Fourth Hospital of Jinan City, Taishan Medical College, Jinan, China; 4 Clinical Laboratory, Yantaishan Hospital, Yantai, China; 5 Laiwu Hospital, Shandong, China; 6 School of Medicine, Shanghai Jiao Tong University, Shanghai, China; 7 Institutes of Biomedical Sciences, Fudan University, Shanghai, China; IPO, Inst Port Oncology, Portugal

## Abstract

Genome-wide association studies (GWAS) have identified several genetic susceptibility loci for breast cancer (BC). One of them, conducted among Chinese women, found an association of rs2046210 at 6q25.1 with the risk of BC recently. Since then, numerous association studies have been carried out to investigate the relationship between this polymorphism and BC risk in various populations. However, these have yielded contradictory results. We therefore performed a meta-analysis to clarify this inconsistency. Overall, a total of 235003 subjects based on 13 studies were included in our study. Significantly increased BC risk was detected in the pooled analysis [allele contrast: OR = 1.13, 95%CI = 1.10–1.17, *P(Z)* <10^−5^, *P(Q)* <10^−4^; dominant model: OR = 1.21, 95%CI = 1.14–1.27, *P(Z)* <10^−5^, *P(Q)* <10^−4^; recessive model: OR = 1.18, 95%CI = 1.12–1.24, *P(Z)* <10^−5^, *P(Q) = *0.04]. In addition, our data revealed that rs2046210 conferred greater risk in estrogen receptor (ER)-negative tumors [OR = 1.27, 95%CI = 1.15–1.40, *P(Z)* <10^−5^, *P(Q)* <10^−4^] than in ER-positive ones [OR = 1.18, 95%CI = 1.09–1.28, *P(Z)* <10^−4^, *P(Q) = *0.0003]. When stratified by ethnicity, significant associations were found in Caucasian and Asian populations, but not detected among Africans. There was evidence of heterogeneity (*P*<0.05), however, the heterogeneity largely disappeared after stratification by ethnicity. The present meta-analysis demonstrated that the rs2046210 polymorphism may be associated with increased BC susceptibility, but this association varies in different ethnicities.

## Introduction

Breast cancer (BC) is the most common cancer among women worldwide [1], which is also a highly heterogeneous disease with wide differences in incidence and mortality across ethnicities [2]. It is reported that African American women have a lower incidence of BC than white women in US [3]. However, they have higher BC mortality rates than white women, due to the fact that African American women are more likely to be diagnosed with BC at a more advanced stage. Although the detailed pathogenesis has not been completely elucidated yet, a number of studies have suggested an important contribution of genetic factors in the development of BC [4]–[6]. In the past decades, high-penetrance genes, such as *BRCA1* and *BRCA2*, have been identified as risk factors for BC, but only account for less than 5% of overall BC cases [7]. Recently, several genome wide association studies (GWAS) and replicated studies have reported multiple novel common genetic variants which are associated with BC susceptibility [8]–[14]. The study by Zheng et al. [14] identified a single nucleotide polymorphism (SNP) rs2046210 at 6q25.1 on the increased risk of BC. Each copy of the minor allele of this SNP was estimated to confer an Odds Ratio (OR) of 1.29 among Chinese women and the authors reported a stronger association with estrogen receptor (ER)-negative than ER-positive BC. Moreover, a similar, although weaker, association was also found for this polymorphism in Caucasian women in the same study. There are several genes located near the SNP rs2046210, including the *ESR1* gene, which encodes estrogen receptor α (ERα) and may be involved in the development of BC. Therefore, numerous studies have been carried out to investigate this susceptibility subsequently. However, a proportion of them have produced conflicting results, which may be caused by the restriction of sample size or ethnic diversity. And individual studies may have insufficient power to achieve a reliable conclusion.

To clarify this inconsistency, we therefore performed the first comprehensive meta-analysis to determine the overall effect of the rs2046210 polymorphism on the risk of BC.

## Materials and Methods

### Literature Search

Eligible literatures in English language published before January 10th 2013 were identified by a search of PUBMED, EMBASE and ISI Web of Science using combination of following keywords: breast cancer, 6q25.1, rs2046210, single nucleotide polymorphism (SNP or polymorphism). All the references cited in these studies and previous review articles were reviewed to identify additional relevant publications.

### Inclusion Criteria

For inclusion, eligible studies had to meet all of the following criteria: (1) the studies were published in peer-reviewed journals and were original papers having independent data from other studies; (2) the studies investigated the association between rs2046210 and BC risk using either case–control or cohort design; (3) the studies have sufficient data to calculate the odds ratio (OR) with its 95% confidence interval (CI) and *P*-value; (4) the studies described the genotyping methods, equipment and protocol used or provide corresponding reference to them; (5) the diagnosis of BC patients was confirmed histologically or pathologically. The major reasons for exclusion of studies were (1) used overlapping samples; (2) were case-only studies or review articles.

### Data Extraction

For each study, the following data were extracted independently by two participants: first author’s surname, year of publication, diagnosis criteria, age, ethnicity, study site, study strategy, Hardy-Weinberg equilibrium (HWE) status, genotyping method, source of control, estrogen receptor (ER) status, total number of cases and controls, and genotype frequency among cases and controls. The results were compared and disagreements were discussed and resolved by consensus.

### Statistical Analysis

The chi-square (χ^2^) test was used to test for deviation from Hardy-Weinberg equilibrium (HWE). Odds ratio (OR) with its 95% confidence interval (CI) was used to assess the strength of association between rs2046210 and BC risk. We used allele contrast (A vs. G), dominant (AA+AG vs. GG) and recessive (AA vs. GG+AG) genetic models to examine the relationship between risk allele or genotypes and BC susceptibility. Cochran’s Chi square-based *Q* statistic test was performed to assess possible heterogeneity across studies. If heterogeneity existed (*P*<0.05), the random effects model was adopted to calculate the overall OR value [15]. Otherwise, the fixed effect model was used [16]. 95% CI was constructed using Woolf’s method [17]. The significance of the overall OR was determined by the *Z* test.

Subgroup analyses were performed according to ethnicity (Caucasian, Asian and African populations), ER-positive/negative status, sample size (<5000 and ≥5000), and source of controls. In addition, sensitivity analysis was performed to assess the stability of the results by removing each study in turn from the total and re-analyzing the remainder. Funnel plot and Egger’s regression test were used to assess publication bias. All *P* values were two tailed and *P*<0.05 was considered statistically significant. All the analyses were carried out by means of the Review Manager software package v.5.0 (The Cochrane Collaboration, Oxford, England).

## Results

### Characteristics of Studies

The literature search yielded 127 references, and the study selection process is shown in Figure S1. A total of 13 studies [14], [18]–[29] were finally included in the present meta-analysis with 117372 cases and 117631 controls. Among them, the study by Zheng et al. [14] was calculated only in the subgroup analysis stratified by ER-positive/negative status to make sure that no overlapping samples were used. In terms of ethnicity, 3 studies involved Caucasian subjects, 3 involved Asian subjects, and 3 involved African subjects. In addition, four papers contained information about distinct independent populations, and was analyzed separately. There are 2 hospital-based studies, and six population-based ones, while another five studies used both sources of controls, therefore, were defined as “Mixed” in the subgroup analysis. The characteristics of studies included in this meta-analysis were summarized in Table 1.

**Table 1:** Characteristics of the studies included in the meta-analysis.

Study	Year	Ethnicity	Genotyping method	No. of case/control	Control Source	HWE status
Antoniou [18]	2011	Caucasian	Taqman and iPLEX	8896/8109	Mixed	Yes
Cai [19]	2011	Asian, Caucasian and African-American	Taqman, Affymetrix SNP array, Sequenom and iPLEX	11901/12049	Mixed	No
Campa [20]	2011	Caucasian	Taqman	8298/11543	PB	Yes
Chan [21]	2012	Asian	Dynamic Array and Taqman	1173/1417	HB	Yes
Han [22]	2011	Asian	Taqman	3251/3493	PB	Yes
Hein [23]	2012	Asian and Caucasian	Taqman	56281/51428	Mixed	Yes
Huo [24]	2012	African	Illumina GoldenGateplatform	1059/1383	Mixed	Yes
Jiang [25]	2011	Asian	SNaPshot SNP assay	492/510	PB	Yes
Mulligan [26]	2011	Caucasian	Taqman and iPLEX	7744/6826	HB	Yes
Ruiz-Narvaez [27]	2012	African-American	iPLEX	1149/1841	PB	Yes
Stacey [28]	2010	Asian, Caucasian and African-American	Nanogen Centaurus assay and sequencing	10176/13286	Mixed	NA
Zheng [14]	2009a	Asian and Caucasian	Affymetrix SNP array	6472/3962	PB	Yes
Zheng [29]	2009b	African-American	Sequenom	810/1784	PB	Yes

NA, Not Available.

Mixed, population and hospital-based; PB, population-based; HB, hospital-based.

### Main Results of Meta-analysis

**Figure 1:**
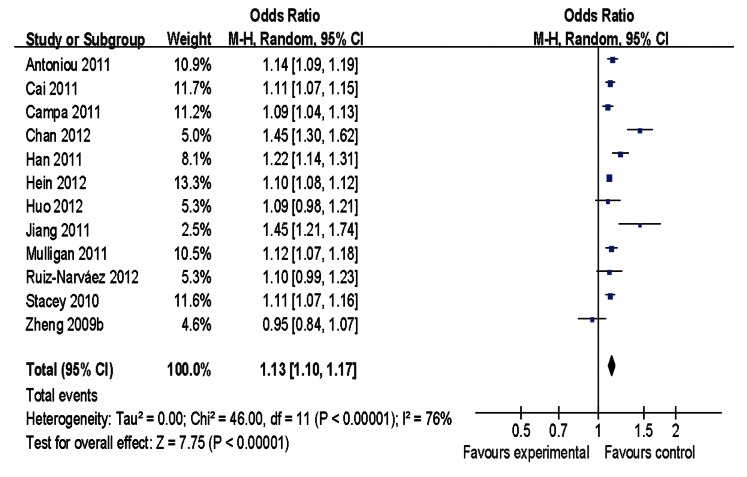
Meta-analysis of rs2046210 and breast cancer risk (allele contrast).

**Figure 2:**
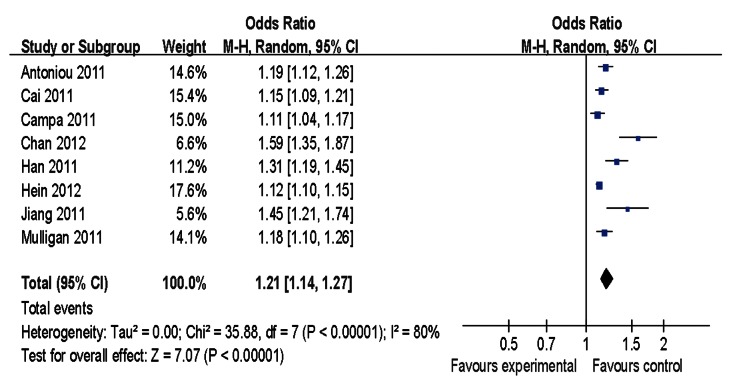
Meta-analysis of rs2046210 and breast cancer risk (dominant model).

**Figure 3:**
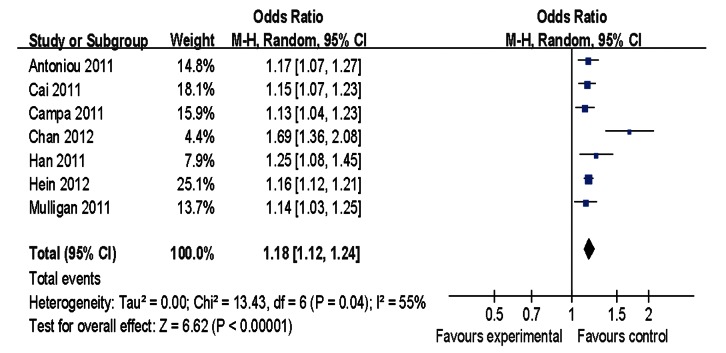
Meta-analysis of rs2046210 and breast cancer risk (recessive model).

Main results of our study investigating the effect of the rs2046210 polymorphism on BC risk are listed in Table 2. Significant associations were found in the pooled analysis between this polymorphism and increased risk of BC [allele contrast: OR = 1.13, 95%CI = 1.10–1.17, *P(Z)* <10^−5^, *P(Q)* <10^−4^; dominant model: OR = 1.21, 95%CI = 1.14–1.27, *P(Z)* <10^−5^, *P(Q)* <10^−4^; recessive model: OR = 1.18, 95%CI = 1.12–1.24, *P(Z)* <10^−5^, *P(Q) = *0.04] as shown in Figure 1–3. When stratified by ethnicity, significant estimations were observed for Caucasians [allele contrast: OR = 1.09, 95%CI = 1.06–1.12, *P(Z)* <10^−5^, *P(Q) = *0.03; dominant model: OR = 1.12, 95%CI = 1.10–1.14, *P(Z)* <10^−5^, *P(Q) = *0.10; recessive model: OR = 1.14, 95%CI = 1.11–1.18, *P(Z)* <10^−5^, *P(Q) = *0.99] and Asians [allele contrast: OR = 1.29, 95%CI = 1.25–1.34, *P(Z)* <10^−5^, *P(Q) = *0.09; dominant model: OR = 1.38, 95%CI = 1.31–1.45, *P(Z)* <10^−5^, *P(Q) = *0.22; recessive model: OR = 1.44, 95%CI = 1.34–1.55, *P(Z)* <10^−5^, *P(Q) = *0.11]. However, there was no evidence indicating significant association among African population in any genetic model (Table 2).

**Table 2:** Meta-analysis of rs2046210 on breast cancer risk.

Subgroup analysis	No. of data sets	Allele contrast			No. of data sets	Dominant model			No. of data sets	Recessive model			
		OR(95% CI)	*P(Z)*	*P(Q)*		OR(95% CI)	*P(Z)*	*P(Q)*		OR(95% CI)	*P(Z)*		*P(Q)*
Total	12	1.13 (1.10–1.17)	<10^−5^	<10^−4^	8	1.21 (1.14–1.27)	<10^−5^	<10^−4^	7	1.18 (1.12–1.24)	<10^−5^	0.04	
Ethnicity													
Caucasian	6	1.09 (1.06–1.12)	<10^−5^	0.03	5	1.12 (1.10–1.14)	<10^−5^	0.10	5	1.14 (1.11–1.18)	<10^−5^		0.99
Asian	6	1.29 (1.25–1.34)	<10^−5^	0.09	5	1.38 (1.31–1.45)	<10^−5^	0.22	4	1.44 (1.34–1.55)	<10^−5^		0.11
African	5	1.01 (0.96–1.07)	0.64	0.09	1	0.83 (0.65–1.06)	0.14	NA	1	0.99 (0.83–1.19)	0.93		NA
Sample size													
<5000	6	1.19 (1.05–1.34)	0.004	<10^−4^	3	1.43 (1.26–1.61)	<10^−5^	0.13	2	1.44 (1.07–1.92)	0.02		0.02
≥5000	6	1.11 (1.09–1.12)	<10^−5^	0.75	5	1.13 (1.11–1.16)	<10^−5^	0.28	5	1.16 (1.12–1.19)	<10^−5^		0.96
Control Source													
PB	5	1.13 (1.03–1.25)	0.009	0.0002	3	1.26 (1.08–1.48)	0.004	0.0007	2	1.16 (1.08–1.24)	<10^−4^		0.24
HB	2	1.17 (1.12–1.22)	<10^−5^	<10^−4^	2	1.23 (1.16–1.31)	<10^−5^	0.0008	2	1.21 (1.11–1.32)	<10^−4^		0.0009
Mixed	5	1.11 (1.09–1.12)	<10^−5^	0.80	3	1.13 (1.11–1.16)	<10^−5^	0.21	3	1.16 (1.13–1.20)	<10^−5^		0.95

**Figure 4:**
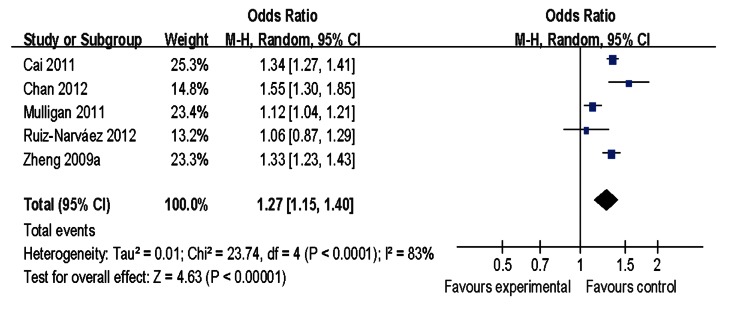
Subgroup meta-analysis of rs2046210 and the risk of ER-negative breast cancer (allele contrast).

**Figure 5:**
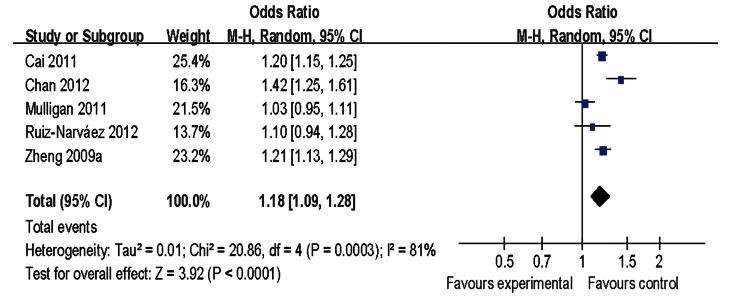
Subgroup meta-analysis of rs2046210 and the risk of ER-positive breast cancer (allele contrast).

We further performed analysis to examine whether this polymorphism was associated with specific prognostic factors by comparing ER-positive cases with ER-negative cases. According to our result, the rs2046210 polymorphism had a stronger association with ER-negative tumors [allelic contrast: OR = 1.27, 95%CI = 1.15–1.40, *P(Z)* <10^−5^, *P(Q)* <10^−4^] than ER-positive tumors [allelic contrast: OR = 1.18, 95%CI = 1.09–1.28, *P(Z)* <10^−4^, *P(Q) = *0.0003] (Figure 4 and Figure 5). When stratified by the source of control, significant estimates were observed among all the subgroups (i.e. population-based, hospital-based and mixed studies) (Table 2). Finally, in the subgroup analysis by sample size, statistically significant associations were found both for studies with large and small sample size (Table 2).

### Sensitivity Analysis and Potential Bias

Each study included in the meta-analysis was removed from the total each time to reflect the influence of individual data set to the pooled ORs. According to the result of sensitivity analysis, the pooled ORs were not qualitatively altered after the removal of any data set, even for two studies by Cai et al. [19] and Stacey et al. [28], which were not in accord with HWE or did not provide detailed information for the calculation. The funnel plot and Egger’s test were performed to assess publication bias of the included literatures. The shape of inverted funnel plots was symmetrical as shown in Figures S2, S3, and S4. Egger’s test also indicated that no publication bias existed (*P*>0.05).

## Discussion

The rs2046210 polymorphism was first identified as a risk variant for BC by Zheng et al [14] among Chinese women. After that, several studies have replicated this association in different Asian populations including Chinese [19], [21], [25], [28], Japanese [19] and Korean [22]. Since minor allele frequencies of SNPs are highly variable and the linkage disequilibrium (LD) patterns differ between ethnicities, it is important to validate the effects of the risk variants identified in GWAS studies in different ethnic populations. Therefore, Zheng et al. [14] evaluated the association in an independent sample of European ancestry in the same study, which have found a similar, although weaker, association. Other studies using Caucasian [18]–[20], [23], [26], [28] or African [19], [24], [27]–[29] subjects have also been performed to further examine the relationship between rs2046210 and BC risk. But some reported conflicting results. To investigate this inconsistency, we provided the first comprehensive meta-analysis to determine the potential relationship between this polymorphism and BC risk, based on 13 studies including 117372 cases and 117631 controls. Our results demonstrated that there was significant association between rs2046210 and increased BC susceptibility.

In the stratified analysis by ethnicity, significant associations were found among Caucasians and Asians in all genetic models, while no association was detected in African population. In fact, the frequency of the risk-associated A-allele was substantially higher in African population (e.g. 69% in HapMap YRI) than in Caucasian (e.g. 29% in HapMap CEU) or Asian populations (e.g. 38% in HapMap CHB). It has been reported that the A-allele may consist of different haplotypes with different risk associations in African population. The study by Ruiz-Narváez et al. [27] showed that rs2046210 was indeed associated with BC susceptibility in African-American women after adjusting for the haplotype background including another SNP rs2046211, which is located in a smaller LD block with rs2046210 in HapMap YRI samples. This may explain the failure to replicate the rs2046210 association with BC risk in African populations in previous studies [19], [24], [28], [29] and in the present meta-analysis. Therefore, additional studies focusing on other loci which are in LD with the rs2046210 polymorphism are warranted to further elucidate the role of ethnic differences in the effect of this SNP on BC risk. Moreover, different lifestyle and environment may also be the reasons for different association results in different populations, due to the fact that these factors may interact with genetic variants to modify the risk of developing cancer [23].

Meta-analysis is often dominated by a few large studies, which may remarkably reduce the evidence from smaller studies. However, in the stratified analysis according to sample size, significantly increased BC risk was found in both large and small studies for all genetic models. In the subgroup analysis based on control source, we also found significant associations in all the subgroups (i.e. population-based, hospital-based and mixed studies), and there was no obvious difference between these subgroups. In addition, our data indicated that rs2046210 was more closely related to ER-negative BC, which is consistent with previous reports in Chinese women [30]. This heterogeneity in the BC risk of different tumor types observed in our study may provide further support for the hypothesis that ER-negative and ER-positive tumors result from different etiologic pathways, rather than different stages of tumor evolution within a common carcinogenic pathway [31].

This polymorphism is located upstream of the *ESR1* gene (29 kb upstream of the first untranslated region and 180 kb upstream of the first coding exon) [32]. *ESR1* encodes estrogen receptor α (ERα), which regulates signal transduction of estrogen, a sex hormone that plays a central role in the etiology of BC. Elevated estrogen levels have been reported to be associated with increased BC risk [33]. Because the biological effects of estrogen are mediated primarily through its high affinity binding to ERs, genetic variants in ER genes, including *ESR1* and *ESR2*, have been the focus of numerous epidemiologic studies on BC risk [34]–[36]. Given its relatively close location to *ESR1*, the rs2046210 polymorphism or SNPs in LD with it may modulate the expression of *ESR1* and alter its downstream signaling, and then is likely to affect BC susceptibility. Interestingly, it is found in a recent GWAS study that the 6q25.1 locus is associated with bone mineral density, which is also a phenotype affected by estrogen [37].

Despite our efforts to investigate the relationship between rs2046210 and BC risk, some limitations should be acknowledged. First, the subgroup meta-analysis examining the interaction between the ER status and the effect of rs2046210 on BC susceptibility was performed based on limited studies with such information available. Therefore, selection bias may have occurred and the corresponding result may be over inflated. In this context, more reliable result can be expected if relevant statistical data are available for a pooled analysis. Second, results in this meta-analysis were based on unadjusted estimates. If all the individual raw data were provided, a more precise analysis could be conducted which would allow for the adjustment by other covariates including age, drinking status, cigarette consumption and other lifestyles. Third, there was substantial between-study heterogeneity found in the overall pooled analysis. However, the heterogeneity was effectively decreased after stratification by ethnicity. In spite of these limitations, no publication bias was observed, and the large number of study subjects still guarantees the statistical power of our analyses.

To conclude, this meta-analysis showed that the rs2046210 polymorphism was significantly associated with increased rick of BC, particularly in Caucasian and Asian populations. Further studies with more accurate phenotype and genotype data, detailed individual information, larger sample size of different ethnic populations and standard statistical methods will be needed to validate our results. Moreover, the interactions between rs2046210 and other SNP loci which are in LD with it should also be evaluated to further elucidate the underlying relationship between this polymorphism and the risk of BC.

## Supporting Information

Figure S1 **Flow diagram of the literature selection process.**
(DOCX)

Figure S2 **Funnel plot of the association between rs2046210 and breast cancer risk (allele contrast).**
(DOCX)

Figure S3 **Funnel plot of the association between rs2046210 and breast cancer risk (dominant model).**
(DOCX)

Figure S4 **Funnel plot of the association between rs2046210 and breast cancer risk (recessive model).**
(DOCX)

PRISMA Checklist S1 (DOC)
