# Supplementary figures and images for: Quantitative Assessment of the Association between rs2046210 at 6q25.1 and Breast Cancer Risk
Source: PLoS One. 2013 Jun 13;8(6):e65206. doi: 10.1371/journal.pone.0065206 (PMC3681980; doi:10.1371/journal.pone.0065206)

Figure S1. Flow diagram of the literature selection process.


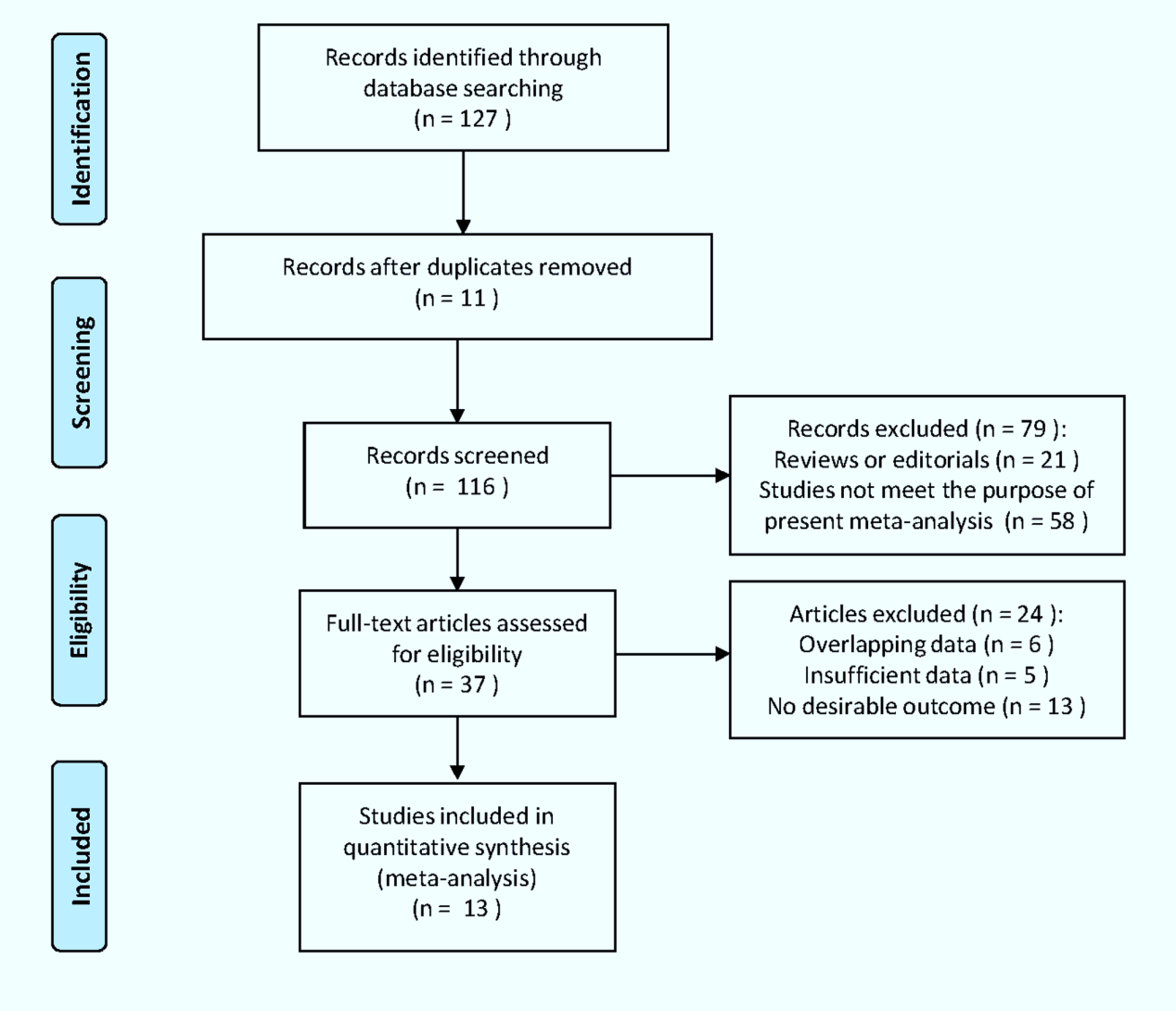

Supplement: Figure S1 — Flow diagram of the literature selection process. (DOCX) [file pone.0065206.s001.docx]

Figure S2. Funnel plot of the association between rs2046210 and breast cancer risk (allele contrast).


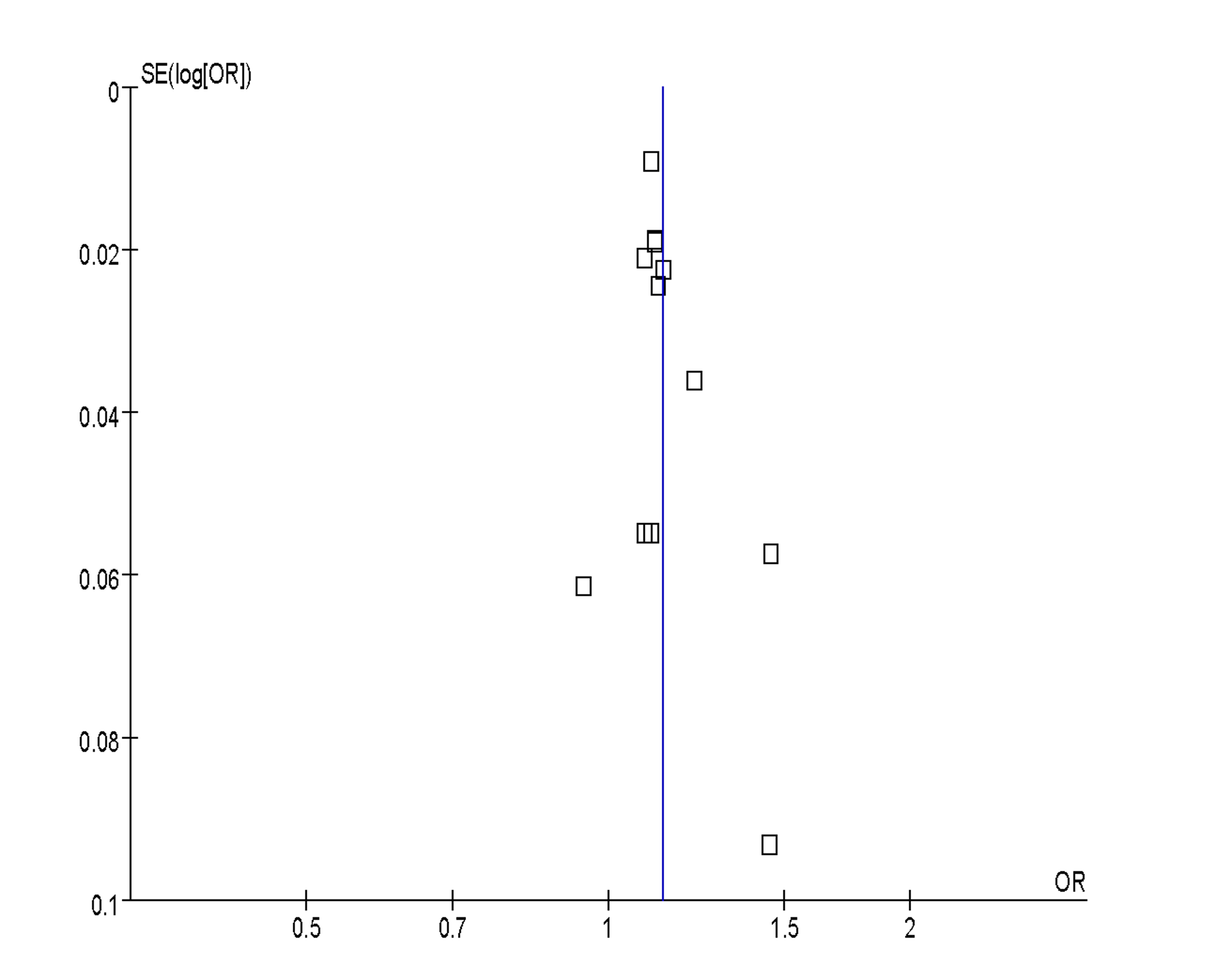

Supplement: Figure S2 — Funnel plot of the association between rs2046210 and breast cancer risk (allele contrast). (DOCX) [file pone.0065206.s002.docx]

Figure S3. Funnel plot of the association between rs2046210 and breast cancer risk (dominant model).


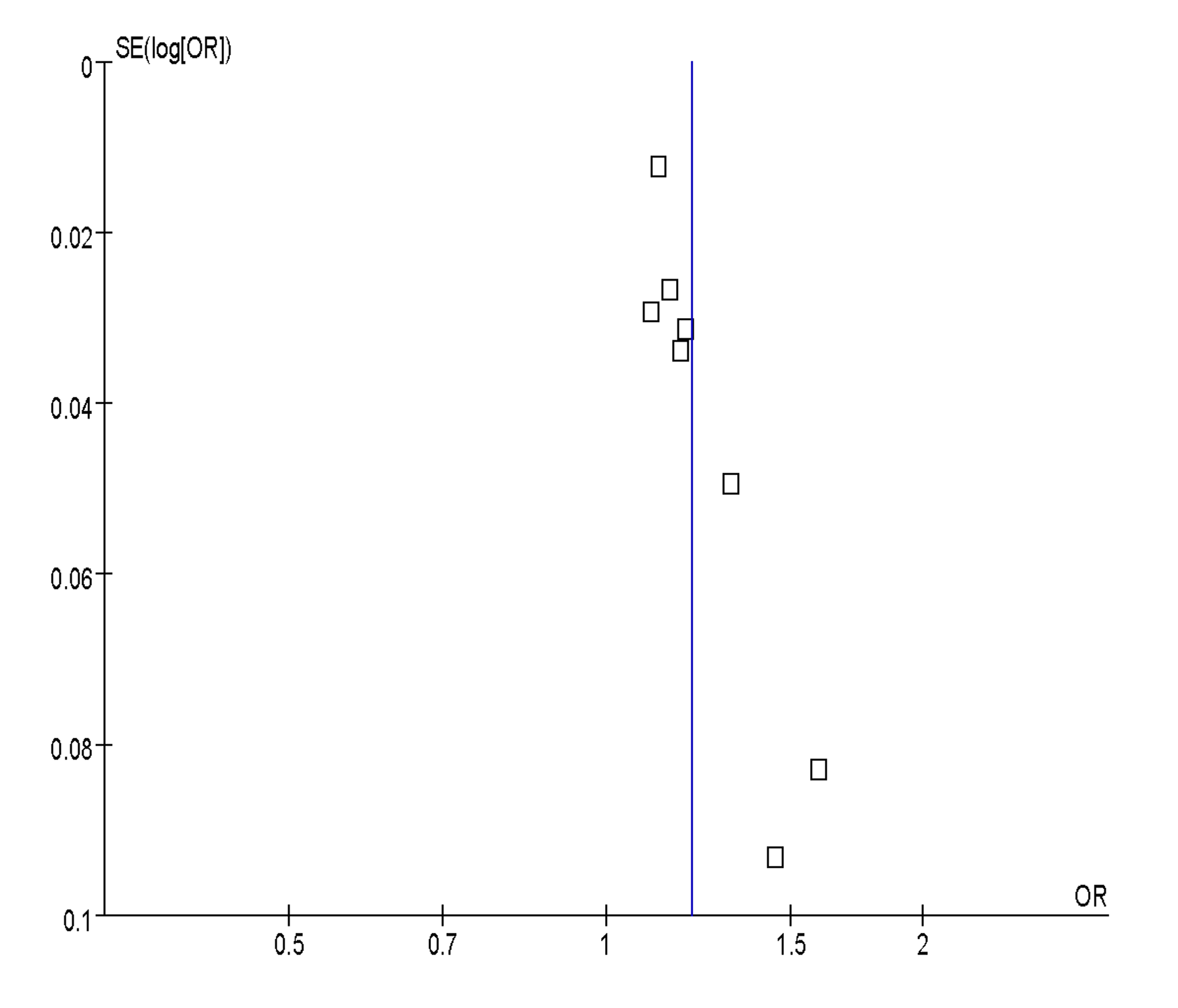

Supplement: Figure S3 — Funnel plot of the association between rs2046210 and breast cancer risk (dominant model). (DOCX) [file pone.0065206.s003.docx]

Figure S4. Funnel plot of the association between rs2046210 and breast cancer risk (recessive model).


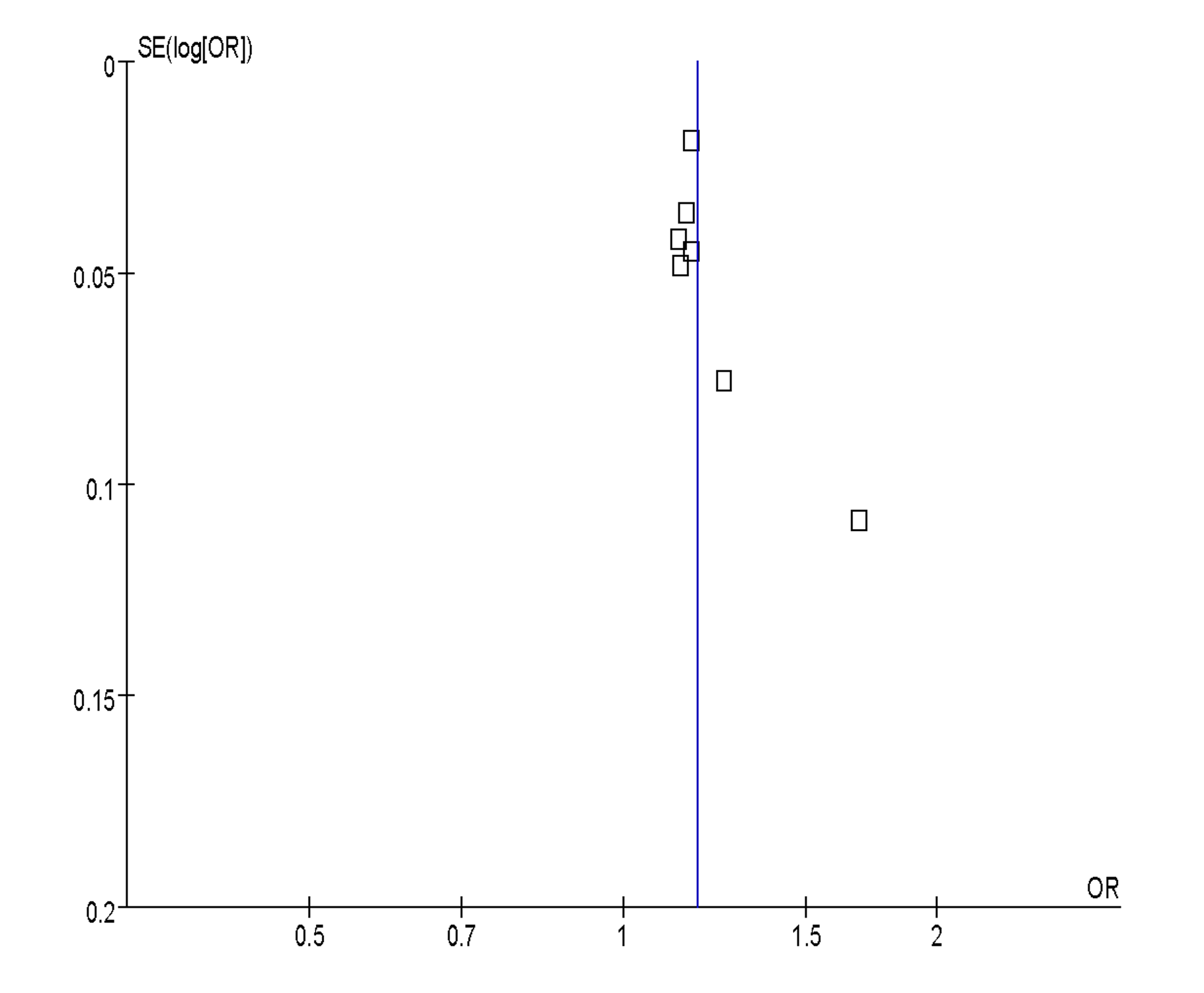

Supplement: Figure S4 — Funnel plot of the association between rs2046210 and breast cancer risk (recessive model). (DOCX) [file pone.0065206.s004.docx]
